# Organic parasite control for poultry and rabbits in British Columbia, Canada

**DOI:** 10.1186/1746-4269-7-21

**Published:** 2011-07-14

**Authors:** Cheryl Lans, Nancy Turner

**Affiliations:** 1 PO Box 72045 Sasamat, Vancouver, British Columbia, V6R4P2, Canada; 2 University of Victoria, School of Environmental Studies, British Columbia, V8W 2Y2, Canada

**Keywords:** poultry, rabbits, ethnoveterinary medicine, ectoparasites, endoparasites, British Columbia

## Abstract

Plants used for treating endo- and ectoparasites of rabbits and poultry in British Columbia included *Arctium lappa *(burdock), *Artemisia *sp. (wormwood), *Chenopodium album *(lambsquarters) and *C. ambrosioides *(epazote), *Cirsium arvense *(Canada thistle), *Juniperus *spp. (juniper), *Mentha piperita *(peppermint), *Nicotiana *sp. (tobacco), *Papaver somniferum *(opium poppy), *Rubus *spp. (blackberry and raspberry relatives), *Symphytum officinale *(comfrey), *Taraxacum officinale *(common dandelion), *Thuja plicata *(western redcedar) and *Urtica dioica *(stinging nettle).

## 1. Introduction

Consumers, butchers and restaurant-owners are increasingly demanding that meat animals be reared in environmentally-sensitive ways that also take animal welfare concerns into consideration (e.g. access to pasture); these organic farming management practices also improve meat quality [1-5]. The meat from poultry and rabbits is more efficient to produce in terms of land use, feed and water use than beef and pork and thus produces a lower environmental impact [6-10]. Some consumers are also concerned about chemical residues (like flubendazole) in meat [11,12]. The access to pasture demanded by animal welfare agents increases the need for parasite control in food animals [11,13]. Organic agriculture allows a restricted number of substances to be used for pest control.

Some conventional livestock farmers add subclinical levels of antibiotics to the animal feed of millions of food animals as growth promoters [14]. Some of these antibiotics are not absorbed and are excreted in manure which is then applied as a fertilizer to food crops. As much as 387 g of chlortetracycline and 202 g of tylosin per hectare is estimated to be added to the soil with the application of pig manure. Greenhouse studies conducted on corn (*Zea mays *L.), green onion (*Allium cepa *L.), and cabbage (*Brassica oleracea *L. Capitata group) showed that all three crops absorbed chlortetracycline from pig manure but tylosin was not absorbed [14]. Botanical and mineral products used for animal health are less likely to become soil contaminants than chlortetracycline since they are natural products.

Extracts and essential oils of various plants such as *Rosmarinus officinalis *L. (rosemary), *Mentha piperita *L. and *M*. *virdis *(L.) L.(mints), *Artemisia absinthium *L. (absinthium, or wormwood), *Chenopodium ambrosioides *L. (epazote), *Thymus vulgaris *L. (thyme) and *Origanum vulgare *L. (oregano) have potential for use as parasite controls because they have insecticidal activity. For example, essential oils of *Melissa officinalis *L. (0.12%) and *Mentha piperata *L. (1.3%) caused mortality and induced repellency in adult females of the carmine spider mite (*Tetranychus cinnabarinus *Boisd.; Acarina: Tetranychidae) and egg-laying was reduced [15]. Nymphs of cayenne tick (*Amblyomma cajennense *(Fabricius); Acari: Ixodidae) were susceptible to ethanolic extracts of *Chenopodium ambrosioides *[16].

### 1.2. Ethnoveterinary research

Ethnoveterinary medicinal research is often undertaken as part of a community-based approach that serves to improve animal health and provide basic veterinary services in underserved areas [17]. This paper reports on research that documented and validated (in a non-experimental way) ethnoveterinary medicines used for parasite control by small-scale, organic livestock rabbit and poultry farmers in British Columbia (B.C.), Canada.

### 1.3. Organic rabbit and poultry production

Most rabbits are raised on farms for home consumption or for the pet industry. In 2001 over 17,000 rabbits on 264 farms were reported; this figure represented a 14% decline in the number of rabbits and a 71% decline in the number of farms with rabbits [18]. There were no certified organic rabbit producers in British Columbia and only two in Canada [19,20].

In the case of poultry, organic egg production represented less than 2 percent of all egg production in B.C. and less than half of 1 percent across Canada [21]. Small-scale farmers produce 100,000 chickens annually. Flocks containing less than 99 layers, or 199 broilers, are not covered by the provincial quota and are not regulated by the provincial Egg or Chicken Marketing Boards.

There are three categories of specialty chicken regulated by the Provincial Marketing Boards. These categories contain 3.6% of the total permit allocation for all chicken production in B.C. (1,085,005 kg live weight) [22]. There were 19 farmers in the specially-fed/housed chicken category, holding half of the allocated permits; 16 of the 19 farmers were located in the Lower Mainland area of B.C. This specially-fed/housed category includes vegetable-fed chickens, chickens raised with no or limited antibiotic use, and chickens classified as organic, certified organic, natural, range fed, SPCA certified, Cornish and roasters [22]. There were also 19 small-scale farmers in the farm gate category, and 20 Lower Mainland farmers in a third category who raised Asian specialty chickens (e.g. Silkies, Taiwanese). The annual economic value of the specialty chicken industry was estimated at $18.0 million dollars, or 7.7% of the entire economic value of all chicken production in B.C. ($232.7 million in 2002); 54.9% of the value of broiler hatching egg production of $32.8 million and 20% of the economic value of egg production of $90 million [22].

## 2. Materials and methods

### 2.1. Data collection

Ethnoveterinary data for British Columbia was collected for a wide range of animals who were under the care of farmers, veterinarians and animal care specialists over a six-month period in 2003. All of the available literature about livestock farmers and the secondary literature on ethnomedicinal plants, folk medicine and related fields in British Columbia was reviewed [23,24]. The research area included south Vancouver Island, the Lower Mainland, and the Thompson/Okanagan region of the Interior. A purposive sample of livestock farmers was used to find 60 key informants. Participants were identified from membership lists of organic farmers, horse and dog breeders and trainers, horse stables, sheep, cattle and goat breeders, naturopaths, farm women's networks, meat processors, holistic veterinarians and other specialists in alternative medicine for animals. Ten farmers (nine poultry and one rabbit farmer), and three herbalists provided the data presented here on plants used for poultry and rabbit parasite control. The participating poultry farmers were either organic (commercial operations) or farm-gate producers, including one also raising Asian specialty chickens.

**Figure 1:**
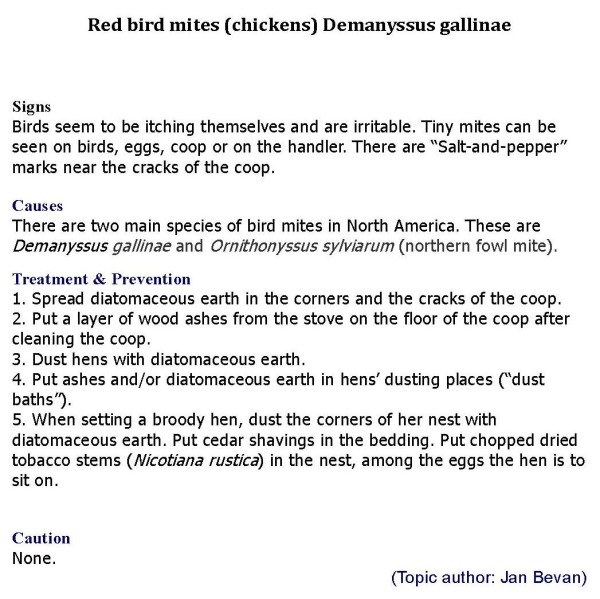
**Data collection form**.

Two visits were made to each farm or respondent, with interviews conducted on the first visit to identify the ethnoveterinary remedies known to and/or used by the individual. The data form was revised on campus and then posted to the relevant address and followed up with a phone interview or a second visit to re-confirm the accuracy of the data (Figure 1). During the second visit, the data recorded and summarized from the initial interview was checked and elaborated on, in order to establish that dosages were accurately noted, for input on content, and to clarify any points. The respondent-approved data forms were compiled into a draft manual.

Where possible, voucher specimens of plants established as remedies were collected by two student ethnobotanists and two herbalists, identified, processed and labelled, then deposited in the University of Victoria herbarium.

The plant-based remedies were evaluated for safety and efficacy with a non-experimental method, prior to including them in the final version of the manual. Published sources such as journal articles, books, and databases on pharmacology and ethnomedicine available on the Internet were searched to identify the plants' known chemical compounds and clinically tested physiological effects. This data was incorporated with data on the reported folk uses of the plants, and their preparation and administration in North America and Europe. For each species or genus the ethnomedicinal uses in other countries was noted, followed by a summary of chemical constituents, as well as any known active compounds. This type of ethnopharmacological review and evaluation is based on previous work [25]. The non-experimental validation of the plants is provided in the discussion section of this paper.

### 2.2. Validation workshop

The International Institute of Rural Reconstruction (IIRR) developed the workshop method used in this research [26]. The workshop process results in the selection of ethnoveterinary practices and remedies that can be effectively recommended for use by the general public and farmers to alleviate minor diseases and problems in domesticated animals.

**Figure 2:**
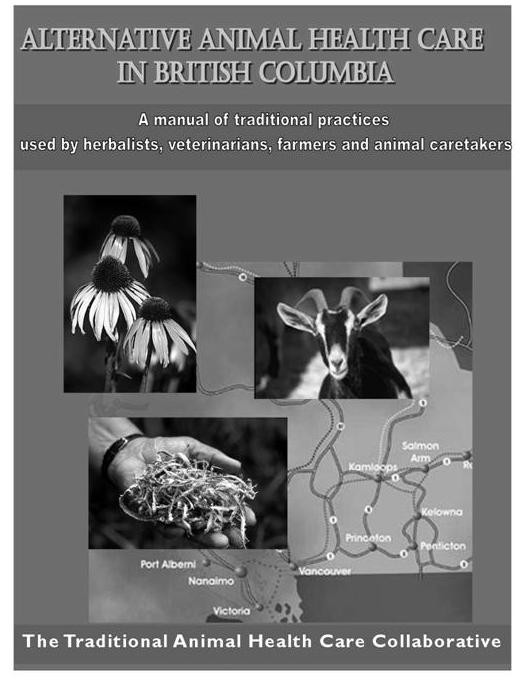
**Manual given to all research participants (180 pages)**.

Ten participants with experience in traditional human and ethnoveterinary medicine took part in a participatory five-day-long workshop hosted by the first author and a German ethnoveterinary consultant (Dr. Evelyn Mathias) in October 2003. Two editorial assistants/facilitators also participated. The facilitators asked participants very specific questions about the medicinal plants used [26]. Each animal/livestock species was covered in a morning or afternoon session. At the poultry session there were three farmer participants and one herbalist, who were already acquainted with the participatory workshop method from the previously-held ruminant workshop. They reviewed collectively the previously prepared draft manual on poultry and rabbits that was in turn based on the earlier one-on-one interviews. Guided by the discussions, the poultry and rabbit data was further clarified, edited and included in the user-friendly manual with the information on other livestock species [27] (Figure 2). There was no separate discussion for rabbits.

## 3. Results

Nineteen plants from 12 plant families were documented as used for poultry parasite control, and 11 plants from eight families were used for parasite control in rabbits (Table 1). Most of these plant species are introduced weeds or kitchen herbs. Some details of how preparations were made are outlined below.

**Table 1:** Ethnoveterinary medicine used for poultry and rabbits in British Columbia

Scientific name, (botanical family) Voucher specimen number	Local name	Part(s) used	Ethnoveterinary use
*Acer macrophyllum *Pursh (Aceraceae) JB 043	big-leaf maple	leaves	bedding - poultry & rabbits

*Symphytum officinalis *L. (Boraginaceae) JBCL 08	comfrey	fed fresh or dried leaves	diarrhoea poultry

*Galium aparine *L. (Rubiaceae) JB107	cleavers	fresh or dried leaves and stems	diarrhoea poultry

*Papaver somniferum *L. (Papaveraceae) JB 114	poppy	fresh leaves or green pods	diarrhoea poultry

*Echinacea purpurea *(L.) Moench (Asteraceae) JBCl 07	Echinacea	leaves are chopped and fed	disease prevention chicks

*Fucus vesiculosus *L. (Fucaceae; Brown Algae) JBCL 11	Kelp	meal added to feed bins every two weeks	disease prevention chicks

*Ulmus fulva *Muhl. (Ulmaceae) not collected	slippery elm	Bark powder in feed for first two weeks	disease prevention chicks

*Linum usitatissimum *L. (Linaceae) not collected	Flax	Ground seed	Starter ration chicks

*Ascophyllum nodosum *(L.) Le Jolis (Fucaceae) not collected	Norwegian sea kelp	Dried meal	Starter ration chicks

*Chenopodium ambrosioides *L. (Chenopodiaceae) JBR 36	Epazote	whole plant	Endoparasites poultry

*Chenopodium album *L. (Chenopodiaceae) JBR 94	Lamb's quarters	whole plant	Endoparasites poultry

*Artemisia vulgaris *L. (Asteraceae) JB 108	Mugwort	whole plant	Endoparasites poultry

*Arctium lappa *L. (Asteraceae) CR 100	common burdock	whole plant	Endoparasites poultry

*Symphytum officinalis *L. (Boraginaceae) CR 35	Comfrey	whole plant	Endoparasites poultry

*Taraxacum officinale *Weber (Asteraceae) CR 46	common dandelions	whole plant	Endoparasites poultry

*Mentha piperita *L. (Lamiaceae) SS024	peppermint	whole plant	Endoparasites poultry

*Cirsium arvense *(L.) Scop. (Asteraceae) SS030	wild Canada thistle	whole plant	Endoparasites poultry

*Urtica dioica *L. (Urticaceae) SS023	stinging nettle	whole plant	Endoparasites poultry

*Rubus spectabilis *Pursh (Rosaceae) JB038	salmonberry	whole plant	Endoparasites poultry

*Nicotiana rustica *L. (Solanaceae) not collected	wild tobacco	handful of crumbled dry leaves or decoction	endoparasites poultry

*Nicotiana rustica *L. (Solanaceae) not collected	wild tobacco	chopped stems, seed pods and leaves	external parasites poultry

*Nicotiana rustica *L. (Solanaceae) not collected	wild tobacco	chopped dried stems	red bird mites

*Thuja plicata *Donn ex D. Don (Cupressaceae) JBR 21	western red-cedar	shavings	red bird mites

### 3.1. Leg mites (Cnemidocoptes mutans)

One cup [250 mL] of cooking oil (e.g. canola or dregs of olive oil) was mixed with a few tbsp [~50 mL] of sulphur powder and diatomaceous earth. After stirring well this mixture was rubbed on the birds' legs, or their legs were dipped in the mixture. This procedure was used to suffocate mites.

### 3.2. Internal parasites in poultry

Poultry were given access to growing epazote (*Chenopodium ambrosioides *L.) so that they could nibble it. Alternatively handfuls of epazote were thrown to the chickens while they were stilled penned if ample quantities were available. Lambsquarters (*Chenopodium album *L.) was used as an alternative to epazote and both plants are also considered very nutritious food for poultry. Mugwort (*Artemisia vulgaris *L.) was grown in the fields or pathways of the farm so that birds could self-medicate. Birds were also allowed to self-medicate with the following herbs: burdock (*Arctium lappa *L.), comfrey (*Symphytum officinale *L.), dandelions (*Taraxacum officinale *Weber), peppermint (*Mentha piperita *L.), wild Canada thistle (*Cirsium arvense *(L.) Scop), stinging nettle (*Urtica dioica *L.), and salmonberry shoots and leaves (*Rubus spectabilis *Pursh).

### 3.3. Caecal/cecal worms (Heterakis gallinarum) and blackhead disease (Histomonas meleagridis)

A handful of crumbled dry leaves of wild tobacco (*Nicotiana rustica L*.) (grown on the farm) was added to 1 bucket of feed. Alternatively a strong decoction was made by filling a canning kettle with the leaves of the wild tobacco and then pouring water into the kettle until full. The kettle was simmered for a few days until the mixture was reduced to one-fourth of the original amount. One cup (250 mL) of the resulting decoction was diluted with 1 gallon of water [ca. 5 L] and given as the drinking water to the flock. Five gallons of water (with 5 cups [about 1 L] of the decoction diluted in it) was said to last for five days (depending on the number of birds in the flock).

## 4. Discussion and Conclusion

The non-experimental validation of the plants is presented in Table 2, in alphabetical order of the plants' scientific names. Table 2 also contains the references numbered 28 - 68.

**Table 2:** Non-experimental validation of plants used for parasite control in poultry and rabbits in British Columbia

Medicinal plant	Validation information	Reference
*Acer macrophyllum *	Big-leaf maple leaves were used as bedding for poultry & rabbits, but not specifically to control parasites in the litter. The fallen, dried leaves were raked up in the autumn, and were then stored for use over the year. The leaf litter has more Ca, K, Mg, molybdenum (Mo), and zinc (Zn) than other trees. The litter decomposes quickly and has a high pH. The leaves contain tannins.	[28,29]

*Ascophyllum nodosum*	Norwegian sea kelp (*Ascophyllum nodosum*) was used as a starter ration for chicks that prevented disease. This species, given as a supplement (2% DM) for two weeks prior to slaughter to feedlot steers and heifers (*Bos indicus *x *Bos taurus*) decreased the prevalence of enterohemorrhagic *Escherichia coli *and it may prevent increases in *Salmonella *species. Supplementation of *Ascophyllum nodosum *to a diet of fescue hay enhanced immune function in lambs and protected against prolonged heat-induced oxidative stress. The vitamin content of *Ascophyllum nodosum *is highest in September and February at 500 mg/kg dry matter. The plant has summer antimicrobial activity but none in spring or winter. Maximal calorific values occur in July in the period of maximum growth.	[30-32]

*Arctium lappa*	Common burdock whole plant was used for endoparasites in poultry. Traditionally seeds were used to purify the blood. Seeds contain chlorogenic acid, caffeic acid, cynarin, lappaol C, arctiin, arctignan E, matareisinol, lappaol A and F and Arctigenin. Arctigenin is a lignan with antioxidant and anti-inflammatory activities. Roots and leaves contain chlorogenic acid, caffeic acid, cynarin, quercitrin, arctiin, quercetin and luteolin.	[33-35]

*Artemisia vulgaris*	Mugwort whole plant was used to treat endoparasites in poultry. 300 mg/kg doses of methanol extracts of the aerial parts of *A. vulgaris *and *A. absinthium *were found to reduce the larval form of *Trichinella spiralis *in rats. *Artemisia scoparia *flowers and *Artemisia pallens *essential oil have shown anthelmintic activity. This use is traditional and was part of a compound remedy used to rid the human body of Taenia with Senna, *Spigelia marilandica *or *Artemisia santonica *together with pumpkin seeds and slippery elm bark.	[36-38]

*Chenopodium album *and *Chenopodium ambrosioides*	Lamb's quarters and epazote whole plants were used for endoparasites in poultry. *Chenopodium album *possesses anthelmintic activity *in vitro *and *in vivo *against mature *Haemonchus contortus *and its eggs and was slightly less effective than Levamisole. The traditional infusion of *Chenopodium ambrosioides *used as a vermifuge is safer than using the herb's essential oil.	[39,40]

*Cirsium arvense *	Wild Canada thistle whole plant was used for endoparasites in poultry. This plant contains lignin, callose and silicon. Taraxasterol has moderate anti-inflammatory activity. Tricin-5-*0*-glucoside, Quercetin-3-*O*-rhamnoglucoside, Quercetin-3-*O*-digalactoside, cirsimaritin, pectolinaringen are also found. Some of these compounds have antimicrobial activity	[41-45]

*Echinacea purpurea *	An *Echinacea *product (containing *Echinacea purpurea *(L.) Moench 20,000 mg/40 grams) was added to chicks' feed, or, alternatively, *Echinacea *leaves were chopped and fed to chicks. Echinacea enhances immune function in rats by increasing antigen-specific immunoglobulin production. *Streptococcus pyogenes*, *Hemophilus influenzae *and *Legionella pneumophila *were inactivated by Echinacea. Echinacea aerial and root ethanol extract also reversed the pro-inflammatory responses of *Staphylococcus aureus *(methicillin-resistant and sensitive strains) and *Mycobacterium smegmatis *but had a lesser bactericidal effect.	[46,47]

*Fucus vesiculosus*	Kelp meal was added to the chicks' feed bins every two weeks: 2 cups (about 500 ml) for 300 young birds. Soluble fractions of the marine alga *Fucus vesiculosus *(42.3% yield) are composed of neutral sugars (18.9-48 g/100 g), uronic acids (8.8-52.8 g/100 g), sulfate (2.4-11.5 g/100 g), small amounts of protein (< 1-6.1 g/100 g), and nondialyzable polyphenols (0.1-2.7 g/100 g). The main neutral sugars were fucose, glucose, galactose, and xylose. Sulfated polysaccharides may be natural antioxidants.	[48]

*Galium aparine *	Cleavers fresh or dried leaves and stems were used for diarrhoea in poultry. This plant has traditionally been used for stomach conditions in North America. The insect antifeedant anthraquinone aldehyde nordamnacanthal (1,3-dihydroxy-anthraquinone-2-al) is found in *Galium aparine*.	[35,49,50]

*Juniperus *sp.	(*Dermanyssus gallinae*) red bird mites in poultry were prevented with cedar shavings in the bedding. The antimycobacterial activity of *Juniperus communis *roots and aerial parts was attributed to a sesquiterpene (longifolene) and two diterpenes (totarol and trans-communic acid). Trans-communic acid was not a stable compound in this experiment. Juniper leaf essential oil had some effectiveness against *Dermanyssus gallinae *at 0.14 mg oil/cm(3).	[51,52]

*Mentha piperita*	Peppermint whole plant was used against endoparasites in poultry. Peppermint oil has larvicidal activity against *Aedes aegypti*, *Anopheles stephensi *and *Culex quinquefasciatus *mosquitoes. Methanolic, dichloromethane and hexanic extracts of *Mentha × piperita *had activity against *Giardia lamblia *but an infusion did not.	[53-55]

*Nicotiana rustica*	A handful of the chopped stems, seed pods and leaves of wild tobacco (*Nicotiana rustica *L.) (grown on the farm) was added to the bedding in nest boxes to reduce external parasites. Wild tobacco (handful of crumbled dry leaves or decoction) was used for endoparasites in poultry; the chopped dried stems were used for red bird mites. Anthelmintic activity was found in *Nicotiana tabacum*. Nicotine was used as an insecticide in the past.	[56]

*Papaver somniferum *	Farmers in our study used leaves and plants of opium poppy (*Papaver somniferum*) to treat diarrhoea in their poultry. This implies using the side effects of pain treatment with opioids: hard dry stools and increased gastroesophageal reflux. Activation of mu-opioid receptors by opoids in the gastrointestinal tract is responsible for inhibition of gut motility.	[57]

*Rubus spectabilis *	Salmonberry whole plant is eaten by poultry and said to control endoparasites. This is possibly based on traditional knowledge since *Rubus trivialis *was given for scours in sheep and *Rubus strigosus *infusion was recommended for diarrhoea. Rubus species berries contain bioactive flavonoids, including anthocyanins and proanthocyanidins that promote health.	[35,58,59]

*Symphytum officinale*	Comfrey fed fresh or dried leaves were used for diarrhoea and endoparasites in poultry. The plant is mucilaginous and high in protein. Self-medicating birds apparently did not ingest enough pyrrolizidine alkaloids to be harmed and the content of these alkaloids varies from plant to plant.	[60,61]

*Taraxacum officinale*	Common dandelion (*Taraxacum officinale*) was used by the participants in our study to treat endoparasites in poultry, and as food for both poultry and rabbits. *Taraxacum officinale *pre-treatment (aqueous decoction of dried herb - 10 mg/kg) can reduce the severity of cholecystokinin (CCK)-octapeptide-induced pancreatitis in rats. This plant use is traditional. Many studies conducted on dandelion extracts or its constituents (polyphenolics and sesquiterpenes) from the leaves or roots have shown anti-inflammatory and other activities.	[62-64]

*Thuja plicata *	Western red-cedar shavings were used to protect poultry against red bird mites. *Thuja occidentalis *was tested and found to have some effectiveness against the poultry red mite *Dermanyssus gallinae*.	[65]

*Thuja plicata*	Methanol extracts of western red cedar (commonly used for animal bedding) were tested for antimicrobial activity against anaerobic bacteria and yeast. The test microbes included *Fusobacterium necrophorum*, *Clostridium perfringens*, *Actinomyces bovis *and *Candida albicans *which are found in foot diseases and other infections in animals; the results were not significant. Beta-thujaplicin is a tropolone-related compound purified from the wood of *Thuja plicata*. All *Staphylococcus aureus *isolates were inhibited by beta-thujaplicin with MICs of 1.56-3.13 mg/L. However, a paradoxical zone phenomenon occurred, with each isolate producing regrowth at higher beta-thujaplicin concentrations.	[66,67]

*Ulmus fulva*	Slippery elm bark powder is put in the feed for the first two weeks for disease prevention chicks. This use is traditional.	[35,68]

*Urtica dioica*	*Urtica dioica *was used for endoparasites in poultry in our study. A leaf infusion of *Urtica dioica *L. (2.5 g dry plant leaves infused in 1 L boiled water) protected rats that were given the chemical carcinogen trichloroacetic acid.	[69]

Sulphur is not toxic to mammals and is allowed in pest control in organic agriculture (see http://www.scotland.gov.uk/Publications/2005/05/13153740/37541). *Chenopodium ambrosioides *is one of the plants that are allowed for pest control [70]. Similarly, farm-grown tobacco is allowed for pest control on organic livestock farms even though the nicotine affects acetylcholine receptors in the nervous system [71,72]. A recent study showed that tobacco bio-oil blocked the growth of the bacteria *Streptomyces scabies *and *Clavibacter michiganensis *and the fungus *Pythium ultimum *(all crop pests). The tobacco bio-oil also killed Colorado potato beetles [73]. Some of the plants used to treat poultry and rabbits are also used to treat pets and pigs in British Columbia. Juniper species oil and *Thuja plicata *Donn ex D. Don have been previously reported as flea treatments for pets. Juniper berries were used to treat stomach problems in pets [25,74]. Mugwort (*Artemisia vulgaris *L.) used to treat endoparasites in poultry and pigs; was reported for fly control of pets [74]. Echinacea leaves were used for disease prevention in chicks while Echinacea roots were used to treat microbial infections in pigs [25]. Peppermint (*Mentha piperita *L.) whole plant was used against endoparasites in poultry while the oil was used for stomach problems in pets. Slippery elm (*Ulmus fulva *Muhl.) was fed to chicks for disease prevention and used for stomach problems in pets [25,74].

Table 2 shows that the anti-parasitic and dietary uses of *Arctium lappa L*., *Artemisia *sp., *Ascophyllum nodosum *(L.) Le Jolis, *Chenopodium ambrosioides L*., *Cirsium arvense (L.) Scop*., *Fucus vesiculosus *L., *Galium aparine *L., *Mentha piperita*, *Nicotiana *sp., *Papaver somniferum *L., *Rubus *spp., *Symphytum officinale *L., *Taraxacum officinale *Weber, *Thuja plicata *Donn ex D. Don, *Ulmus fulva *L. and *Urtica dioica *L. are supported by ancient and current scientific studies and reports. For example the essential oils from various plants have shown toxicity to different insect pests. *Artemisia judaica *L., inhibits the normal feeding activity of the cotton leafworm (*Spodoptera littoralis*), *Juniperus occidentalis *Hook, has activity against adult mosquitoes (*A*. *aegypti*), *Xenopsylla cheopis *(rat flea) and *Ixodes scapularis *(tick). *Chenopodium ambrosioides *L., has activity against *Planococcus citri *(citrus mealybug) and western flower thrips (*Frankliniella occidentali*s) [71].

A botanical compound containing *Chenopodium ambrosioides *L., was significantly more effective against green peach aphid, *Myzus persicae *(Sulzer) (Homoptera: Aphididae), western flower thrips, *Frankliniella occidentalis *(Pergande) (Thysanoptera: Thripidae), and greenhouse whitefly, *Trialeurodes vaporariorium *(Westwood) (Homoptera: Aleyrodidae) than neem oil (*Azadirachta indica *A. Juss) and insecticidal soap but was not as effective against the whitefly parasitoid *Encarsia formosa *Gahan (Hymenoptera: Aphelinidae) [70]. Burdock extracts (20 g kg-1) (*Arctium lappa *L.) protected potato leaves from the larvae of Colorado potato beetle (*Leptinotarsa decemlineata *(Say)) [75]. The lyophilized extract of burdock leaves demonstrated antimicrobial activity against some bacteria and fungi (*Bacillus subtilis*, *Escherichia coli*, *Staphylococcus aureus*, *Micrococcus luteus*, *Candida albicans*, *Lactobacillus acidophilus *and *Pseudomonas aeruginosa*) [76]. The essential oil of *Mentha piperita *L., showed activity against *Candida albicans *[77]. *Mentha piperita *L. (methanol and dichloromethane extracts) showed activity against certain yeasts within 24 hours. The most resistant yeasts were *C. glabrata *and *C. utilis*, while *C. krusei *and *C. guilliermondii *were the most susceptible strains [55].

Botanical compound studies on livestock pests have also been conducted (see Table 2). *Thuja occidentalis *L. arborvitae and Juniper spp. (*Juniperus*) leaf essential oils were found to be effective against the poultry red mite *Dermanyssus gallinae *[53,65]; therefore adding these plants to poultry bedding could be recommended. *Mentha longifolia *auct. non (L.) Huds. (synonym *Mentha spicta *L.) ethanol and water extracts had 67.1 and 63.1% efficacy respectively against naturally acquired pinworms (*Syphacia obvelata*) in mice suggesting the usefulness of certain mint species for endoparasite control [78]. *Chenopodium ambrosioides *L., has a long history of use against endoparasites. Mice infected with *Schistosoma mansoni *cercariae were given *Chenopodium ambrosioides *L., methanol extracts at high concentrations (750 and 1000 ppm) and the extracts diminished the cercarial infectivity of the mice [79]. The hexane extract of *C*. *ambrosioides *L., showed anthelmintic activity *in vitro *and a reduction of the inflammatory reaction produced by the infection of *Toxocara canis *larvae *in vivo *in mice and showed no toxicity [80].

The use of opium poppy (*Papaver somniferum L*.) for diarrhoea in poultry was based on its opiate activity; opoids would alleviate diarrhoea by hardening the stools [57].

Kelp was fed to chicks and this practice may improve the food safety of organic poultry. The use of kelp is supported by one study which found that Norwegian sea kelp (*Ascophyllum nodosum *(L.) Le Jolis) given as a supplement (2% DM) for two weeks prior to slaughter to feedlot animals (*Bos indicus *x *Bos taurus*) decreased the prevalence of enterohemorrhagic *Escherichia coli *and it may prove effective in controlling the spread of *Salmonella *species [30].

## Conclusions

This study highlights the potential for local, easily available herbal preparations to be used safely and effectively to treat parasites and various other ailments in animals being raised for meat or other purposes. Nineteen species of plants were used for parasite control in poultry. Eleven species were used for parasite control in rabbits. Plants used for treating endo- and ectoparasites included *Arctium lappa L*. (burdock), *Artemisia *sp. (wormwood), *Chenopodium album *L. (lambsquarters) and *C. ambrosioides *L. (epazote), *Cirsium arvense (L.) Scop*. (Canada thistle), *Juniperus *spp. (juniper), *Mentha piperita *L. (peppermint), *Nicotiana *sp. (tobacco), *Papaver somniferum *L. (opium poppy), *Rubus *spp. (blackberry and raspberry relatives), *Symphytum officinale *L. (comfrey), *Taraxacum officinale *Weber (common dandelion), *Thuja plicata *Donn ex D. Don (western redcedar) and *Urtica dioica *L. (stinging nettle).

Parasitologists have realized that chemoprophylaxis is unsustainable due to increasing drug resistance and the costs of constantly developing new drugs [81]. Certain crop plants can uptake antibiotics from livestock manure applied to the soil. This has implications for human health [14]. The use of botanical products for parasite control would reduce the antibiotic contamination of the soil and lessen the antimicrobial resistance that is developing in certain parasites. Further research is needed to further confirm these preliminary findings on the efficacy and safety of these herbs, but previous studies indicate that their use can be both beneficial and relatively cost effective.

## Competing interests

The authors declare that they have no competing interests.

## Authors' contributions

CL conceived of the study, obtained funding for it and participated in its design and coordination. NT supervised the study, helped hire research assistants, facilitated the Herbarium deposits and took part in the workshop. Both authors read, revised and approved the final manuscript.
